# Muscle Functions and Functional Performance among Older Persons with and without Low Back Pain

**DOI:** 10.1155/2016/8583963

**Published:** 2016-11-02

**Authors:** Nor Azizah Ishak, Zarina Zahari, Maria Justine

**Affiliations:** Department of Physiotherapy, Faculty of Health Sciences, Universiti Teknologi MARA, Puncak Alam Campus, 42300 Puncak Alam, Selangor, Malaysia

## Abstract

This study aims to compare muscle functions and functional performances between older persons with and without low back pain (LBP) and to determine the association between muscle functions and functional performances. This is a cross-sectional study, involving 95 older persons (age = 70.27 ± 7.26 years). Anthropometric characteristics, muscle functions, and functional performances were measured. Data were analyzed using ANOVA, Pearson's correlation, and multiple linear regression. The functional performances showed no significant differences (females LBP versus non-LBP, males LBP versus non-LBP) (*p* < 0.05). For muscle functions, significant differences were found (females LBP versus non-LBP) for abdominal muscle strength (*p* = 0.006) and back muscle strength (*p* = 0.07). In the LBP group, significant correlations were found between back and abdominal muscle strength and hand grip strength (*r* = 0.377 and *r* = 0.396, resp.), multifidus control and lower limb function (*r* = 0.363) in females, and back muscle strength and lower limb function (*r* = 0.393) in males (all *p* < 0.05). Regression analysis showed that abdominal and back muscle strengths were significant predictors of hand grip strength (*p* = 0.041 and *p* = 0.049, resp.), and multifidus control was a significant predictor of lower limb function in females (*p* = 0.047). This study demonstrates that older women with LBP exhibit poorer muscle functions compared to older women without LBP.

## 1. Introduction

Low back pain (LBP) is the most common musculoskeletal problem that affects all age groups. LBP has become a global health concern, because it is the leading cause of disability worldwide [1]. About 60–90% of the population have LBP at least once in their lifetime [2]. In a recent study [3], the prevalence of LBP among older persons living in the community in Ijok, Malaysia, is 25.6%. By contrast, a preliminary study found that 62.5% of older persons living in the institutions suffered from LBP [4]. This evidence shows that the prevalence of LBP among older persons in Malaysia is significant and therefore should not be neglected.

Older persons experience progressive deterioration of the musculoskeletal system that impairs their muscle function, including their strength and control of the lumbar spine muscles. As reported by Singh et al. [5], lumbar extensor strength declines significantly in older persons compared with younger persons. LBP affects an individual's muscle function, especially the back and abdominal muscle strength. Decline in muscle strength as a result of aging may cause functional limitations among older persons, such as walking, kneeling, and performing sit-stand actions.

Back and abdominal muscles maintain spinal stability during body movements. Hirano et al. [6] highlighted the notion that weakness of the back muscle is an important risk factor of locomotion syndrome, which later may lead to limitations in daily activities and quality of life. The authors also revealed that the locomotion syndrome caused by the loss of function of the back muscles leads to walking disabilities. In addition, older adults, who exhibit poor trunk muscle composition and low attenuation indicating higher levels of fat infiltration and less muscle mass, appear to have a greater risk of reduced mobility-related function over time, which is more pronounced in those persons with a history of at least a moderate severity of LBP [7]. The reduction of muscle mass and strength of the trunk muscles may reduce functional mobility among older persons with LBP.

The core muscles, including the multifidus and transverse abdominus (TrA), constitute a kinetic link or a series of segments of the body that transfers torque and angular momentum from the body to the upper and lower limbs during the performance of sport skills, occupational skills, fitness activities, and daily activities [8]. Hodges and Richardson [9] also found that the TrA is the first muscle activated and contracted prior to the movement of the limb. Therefore, during daily activities which include functional mobility, such as walking and chair stand tasks, the TrA and multifidus play important roles in stabilizing the body and ensuring the successful performance of tasks.

LBP patients tend to avoid painful movements of the lumbar joints, which later may reduce the activity of back and abdominal muscles, thereby reducing their strength. Back and abdominal muscle strengths are important for the population with LBP, regardless of age. The weakness of abdominal muscle strength further creates anterior tilting of the pelvis that increases the possibility of having LBP [10] and the intensity of pain. The back and abdominal muscle strength generates and controls the movement of the lumbar joint and provides bracing effect to the spine against compressive force [11]. During activities such as lifting objects, back and abdominal muscles contract to protect the spine against excessive loads acting on it; therefore, these muscles are important for preventing and reducing LBP.

However, limited studies have investigated back and abdominal muscle functions among the elderly. Several studies have investigated the relationship between back and abdominal muscle functions and quality of life [12], spinal mobility [13], and postural balance [14], but none of the studies had focused on the correlation of back and abdominal muscle function and functional performance. These muscle functions may influence the functional performances in older persons, especially for those who have had LBP, yet the association between muscle functions and functional performances has not been studied. The present study aims to (1) compare muscle function (back and abdominal muscle strengths, TrA, and multifidus muscle control) and functional performance among older persons with and without LBP and (2) determine the association between muscle function and functional performance among older persons with LBP. We hypothesize that older persons with LBP may have poorer functional performances and muscle functions compared to older persons without LBP. We also hypothesize that functional performance is significantly correlated with muscle function in the LBP group.

## 2. Methods

### 2.1. Participants and Study Design

This paper presents a cross-sectional study, involving 95 institutionalized older persons (age 60 to 88 years) from four selected public funded institutions in Malaysia. The selections of these homes were based on a preliminary survey identifying those places with a high number of older persons complaining of LBP. The subjects were included in the study based on the following criteria: (1) older persons, aged 60 years and above; (2) having lower back pain/backache/back pain/back disorder, diagnosed by medical doctors; (3) being able to walk independently with or without walking aids; (4) being able to do day-to-day activities independently; and (5) being able to understand and respond to Malay/English language and follow instructions on testing procedures. The subjects were excluded if they presented with the following: (1) permanent disability, comorbidity, waiting for surgery, spinal tumor, senility, dependency most of the time, and serious spinal complication (red flags); (2) diagnosis with mental disorders such as schizophrenia, depression, and delirium; (3) mild cognitive impairment (Mini-Mental State Examination score less than 17) [15]. The Research Ethics Committee of the Faculty of Health Sciences, Universiti Teknologi MARA (UiTM), approved the present study, and permission to conduct the study in the RSK was received from the Social Welfare Department of Malaysia. All subjects who agreed to participate in this study were briefed on the objectives and procedures of the study prior to signing an informed consent form.

### 2.2. Outcome Measures

#### 2.2.1. Anthropometric Data

Anthropometric characteristics such as height (m), weight (kg), body mass index (BMI) (kg/m^2^), waist circumference (cm), and body fat composition (%) were assessed based on standard protocols.

#### 2.2.2. Evaluation of Pain

The pain intensity in the lower back region was measured using the Numeric Rating Scale (NRS). The NRS is a segmented numeric version of the visual analogue scale, where “0” indicates no pain and “10” indicates the worst pain, and subjects choose a whole number (0–10 integers) that best represents the intensity of their pain [16]; higher NRS indicates higher severity of LBP.

### 2.3. Functional Performance

#### 2.3.1. Lower Limb Function

The 30-Second Chair Rise was used to measure the lower limb function and strength, which related to day-to-day activities, for instance, getting out of a chair or climbing stairs. This test required repetitive sit-to-stand movements, which LBP patients might have difficulties in executing. This test is a valid and reliable measure of functional strength and endurance in the lower extremities in older adults [17]. This test required subjects to stand up from a sitting position, sit down again, and repeat the movements in the span of 30 seconds [18]. The cut-off point for this test is 15 repetitions [19], and higher number of sit-stand repetitions shows better lower limb function.

#### 2.3.2. Mobility and Balance

The Timed Up and Go (TUG) test was used to identify balance and mobility among subjects. This test is relatively simple and quick and tests multiple components of balance and mobility, such as sit-to-stand, walking, and turning tasks, which may be difficult for LBP patients. TUG is sensitive to early changes of functional performance [20], which may occur in the presence of LBP. This test had day-to-day stability with ICC of 0.98 and a low standard error of measurement of 0.99 seconds [21]. In the TUG test, subjects sitting on a chair (approximately 46 cm) were required to stand up, walk a 3-meter distance at normal pace, turn, walk back, and sit again [22]. The cut-off time for the TUG test is 13.5 seconds. A result higher than the cut-off time indicates an increased risk of falls among older persons. A shorter time taken to complete the task indicates good mobility and balance.

#### 2.3.3. Hand Grip Strength

The JAMAR hand dynamometer was used to determine the hand grip strength among older persons, because it is easy and useful in identifying the decline of functional performance for hand grip strength, which may decrease in older persons with LBP. The following procedures were performed for the hand grip strength readings [23]. First, subjects were seated with the elbow at 90-degree flexion and the forearm and wrist in neutral position while gripping the dynamometer. Next, they were asked to squeeze the handle of the dynamometer as strong as they can. The measurements were taken with three attempts for both hands with a 1-minute rest in between trials. The cut-off value for hand grip strength is 30 kg for males and 20 kg for females [24], and a higher score indicates greater hand grip strength.

### 2.4. Muscle Functions

#### 2.4.1. Back and Abdominal Muscle Strengths

Abdominal and back muscle strengths were assessed by using a 10 kg mechanical push-pull dynamometer (MPPD). The abdominal and back muscles are superficial muscles that are important in producing and controlling the movement of the trunk [25], such as trunk bending and rotation. The test-retest reliability of MPPD shows that this instrument is reliable, with intraclass correlation ranging from 0.78 to 0.89 [26]. To measure back muscle strength, subjects were in prone lying position and extended their trunk against the MPPD that was placed along the lumbar region. For abdominal muscle strength, subjects were instructed to lift up their body against the MPPD that was placed on their abdomen while in crook lying position [27]. The MPPD's unit of reading is kilograms, and a higher score represents greater back and abdominal muscle strength.

#### 2.4.2. Muscle Control

The muscle control of TrA and multifidus was measured by using pressure biofeedback unit (PBU). The TrA and multifidus increase intra-abdominal pressure that provides stability to the spine [28]. To assess TrA muscle control, subjects were instructed to draw in their abdomen and hold for a 10-second period [29]. The pressure for PBU was set at 70 mmHg, and the pressure reduction readings were recorded. For multifidus muscle control, subjects were asked to draw in their abdomen and maintain it for 10 seconds in crook lying position. The pressure for PBU was set at 40 mmHg, and the pressure reduction readings were recorded. The pressure reduction from 0 to 3 mmHg and 0 to 2 mmHg indicates good and fair muscle control, respectively, and an increase in pressure from the initial pressure indicates poor muscle control [30].

### 2.5. Statistical Analysis

The IBM SPSS statistics software version 20 was used to conduct descriptive statistics and correlation and regression analysis. The normality of data was checked using Kolmogorov-Smirnov, histogram, skewness, and kurtosis. The normality test showed that all of the data were normally distributed. The homogeneity of variance was tested using Levene's test. The mean and standard deviations of all the variables were calculated. The significance level was set at  *p* < 0.05  for each statistical analysis. Power analysis was conducted using GPower 3 software, in which sample size of 76 subjects was sufficient to provide moderate effect. The comparisons of anthropometry characteristics, functional performance, back and abdominal muscle strength, and TrA and multifidus control were analyzed using the one-way analysis of variance (ANOVA). The LBP group was further analyzed by using Pearson's correlation coefficient to determine the association between back and abdominal muscle strength and functional performance domains. Multiple linear regressions were conducted to predict muscle functions and functional performances and force entry method was used in the regression analysis.

## 3. Results

### 3.1. Comparison of Demographic Characteristics, Pain Intensity, Functional Performance, and Muscle Function among Groups

Table 1 shows the demographic characteristics (age, BMI, waist circumference, and body fat composition), pain intensity, functional performance (lower limb function, balance and mobility, and right and left hand grip strength), and muscle function (abdominal and back muscle strength, TrA and multifidus muscle control) among groups.

The mean results for demographic characteristics, as shown in Table 1, indicated that all the variables were not significantly different between the groups with and without LBP, except for body fat composition. In terms of pain intensity among the LBP group, there were no significant differences between males and females (*p* = 1.00). The mean pain intensity for males and females with LBP was 4.14 and 4.13, respectively. The non-LBP group reported no pain in the back region (NRS = 0).

However, for the functional performance, there were no significant differences between males with LBP and without LBP for lower limb functions (*p* = 1.00), balance and mobility (*p* = 0.11), and right and left hand grip strength (*p* = 1.00). On the other hand, there were also no significant differences between females with LBP and without LBP in terms of lower limb function (*p* = 0.718), balance and mobility (*p* = 0.74), right hand grip strength (*p* = 1.00), and left hand grip strength (*p* = 0.718).

In terms of muscle functions, a significant difference was observed between females with and without LBP (*p* = 0.01) for abdominal muscle strength. However, the abdominal muscle strength between males with LBP and without LBP did not show significant differences (*p* = 1.00). In terms of back muscle strength, there was a significant difference between females with and without LBP (*p* = 0.02). In contrast, the back muscle strength for males with LBP and without LBP showed no significant difference (*p* = 1.00). In addition, the muscle control of TrA and multifidus was not significantly different between the LBP and non-LBP groups with  *p* = 0.30  and  *p* = 0.13, respectively. However, the muscle control for both muscles was better in the group with LBP compared with the group without LBP.

### 3.2. Correlation Analysis between Muscle Functions and Functional Performances in LBP Group

Table 2 (Pearson's correlation coefficient) revealed that there were significant correlations between abdominal muscle strength and hand grip strength (*r* = 0.377,  *p* = 0.04) in the female group. However, abdominal muscle strength did not show significant correlation with lower limb function (*r* = 0.10,  *p* = 0.598) or with balance and mobility (*r* = 0.073,  *p* = 0.703). In the male group, insignificant correlation was found between abdominal muscle strength and all domains of functional performance.

In the female group, back muscle strength and hand grip strength positively and significantly correlated (*r* = 0.396,  *p* = 0.03). However, other variables of functional performance did not correlate with back muscle strength in the female group. By contrast, the male group revealed a significant difference between back muscle strength and lower limb function (*r* = 0.393,  *p* = 0.022). Similar to the female group, other variables of functional performance did not correlate with back muscle strength in the male group.

Moreover, no significant correlation between TrA muscle control and all variables of functional performance was found in both groups. Interestingly, in the female group with LBP, multifidus control showed significant and positive correlation with lower limb function (*r* = 0.363,  *p* = 0.049), although other variables of functional performance did not correlate with multifidus muscle control. However, in the male group, no significant correlation between multifidus muscle control and all domains of functional performance was demonstrated.

Table 3 shows the analysis using multiple linear regressions of muscle functions and functional performances. In the group of females with LBP, muscle functions only contributed 30.1% of the variation of the hand grip strength. The abdominal and back muscle strengths were significant predictors of hand grip strength with  *p* = 0.041  and  *p* = 0.049, respectively. However, TrA and multifidus muscle control were not significant predictors of hand grip strength in the group of females with LBP. Muscle functions were insignificant predictors of hand grip strength and only contributed 5% of the variation in the group of males with LBP.

In the group of females with LBP, muscle functions contributed 16.5% of the variation of the lower limb function. Multifidus muscle control was a significant predictor of lower limb function in the females with LBP (*p* = 0.047). However, abdominal and back muscle strength as well as TrA control did not predict lower limb functions. In contrast, in the group of males with LBP, muscle functions were insignificant predictors of lower limb function and only contributed 16.8% of the variation of lower limb function.

In addition, muscle functions were not significant predictors of balance and mobility in both groups. Muscle functions only explained 1.5% and 3.9% of the variation of TUG in the females and males with LBP, respectively.

Table 4 shows the analysis using multiple linear regressions between pain and muscle functions as well as functional performances. Additionally, in the group of females with LBP, the muscle functions and functional performances only contributed 28.4% of the variation of pain intensity, while, in the group of males with LBP, the muscle functions and functional performances explain 22.4% of the variation of pain intensity. Muscle functions and functional performances were insignificant predictors of pain intensity in the low back in both male and female groups (*p* > 0.05). The predictors of pain intensity for older persons with LBP might be explained by other variables.

## 4. Discussion

### 4.1. Comparison of Functional Performances

The present study aimed to compare functional performances between older persons with and without LBP. This study revealed that there were no significant differences in functional performance in terms of lower limb function, balance and mobility, and hand grip strength between older persons with and without LBP.

In terms of lower limb function, our study revealed no significant differences of lower limb function among older persons with and without LBP. The finding of our study was inconsistent with an earlier study by Rudy et al. [31] that discovered significant difference of lower limb function between older persons with chronic LBP and pain-free subjects. However, subjects in Rudy et al.'s study were a mix of the males and females in both groups, while in our study we compared lower limb functions between older persons with and without LBP in both genders. The male subjects may score higher in the lower limb function compared to females, due to their physiological characteristics as they have higher muscle mass than females. Therefore, if the group combines male and female subjects, the lower limb function score in older persons with and without LBP may be inaccurate to reflect their actual score. Another possible reason of insignificant finding is that, despite the presence of LBP, older persons still maintain their functional independence such as stair climbing, getting up from a chair or getting out of bed, maintaining balance, or walking a distance. Therefore, regardless of the presence of pain, older persons are performing functional tasks, making no difference of lower limb function between older persons with and without LBP.

Pain in the back leads to avoidance of activities, leading to disuse, muscle atrophy, and later decreased muscle strength [32]. The reduction of physical activities because of back pain, such as walking, may cause muscle weakening of the lower limbs, which had significant functional consequences on the maintenance of personal independence and the ability to execute daily tasks [33]. Champagne et al. [34] agreed that older persons with chronic LBP demonstrated poorer mobility and balance test compared to non-LBP older persons in the female subjects. However, our study found that balance and mobility were not significantly different between older persons with and without LBP in both male and female subjects. The possible explanation of this unexpected finding is that, despite the presence of LBP, subjects in this study are still walking around at the institutions home, for instance, going to the cafeteria every day. Thus, older persons with LBP maintained the functional activity that required the balance and mobility the same as older persons without LBP.

LBP also was associated with several negative consequences with one of them being decreased physical function [35], including hand grip strength. However, our study demonstrated no significant differences between older persons with and without LBP in the hand grip strength. Although institutionalized older persons perform less or none of the instrumental activities of daily living, such as cooking, washing clothes, and housekeeping, compared with community-dwelling older persons, however, it is not necessary that their hand grip strength is reduced due to the lack of instrumental activities of daily living. Yet, older persons living in the institutions still maintain their basic activities of daily living that utilize their hand functions such as bathing, dressing, grooming, and eating even though they had LBP. Therefore, regardless of whether there is pain or not at the lower back, older persons performed hand functions every day, making no significant difference in hand grip strength in both groups. This indicated that the hand grip strength is not affected much with the presence of LBP.

### 4.2. Comparison of Muscle Functions

The current study also aimed to compare the muscle functions between older persons with and without LBP. It is interesting to note that the abdominal muscle strength was significantly different between older female subjects with and without LBP, but not in males. This indicated that the abdominal muscles strengths of older females were affected with the presence of LBP. Our finding was consistent with a previous study [12] showing that older persons with lumbar osteoarthritis exhibit lower abdominal muscle strength compared with older persons without lumbar osteoarthritis. Although Vieira et al. did not compare the abdominal muscle strengths between each gender, it can be generalized that the abdominal muscle strength between older persons without lumbar osteoarthritis was better than that between older persons without lumbar osteoarthritis. Surprisingly, the abdominal muscle strength in the males with LBP and without LBP did not significantly differ. The observed findings might be due to gender roles. Gender has been associated with the pain response, in which the masculine gender norms in males ordering increased tolerance of pain, while feminine gender norms dictate acceptance of pain [36]. The good coping behavior in males may enable older persons to continue daily activities, thus allowing them to maintain their abdominal muscle strength despite the fact that they have LBP.

It is noteworthy that back muscle strength in the older females with LBP was lower than in older females without LBP, but not in the males group. Contrary to the present study's finding, Bel et al. [37] reported no significant difference in back muscle strength between subjects with LBP and without LBP. However, this study involved a young age group, whose back muscle strength is likely influenced by having active lifestyles. According to Singh et al. [5], the rate of deterioration of back muscle strength among females was two times higher compared with males. In addition, as we explained earlier, higher tolerance of pain in the male subjects enabled older males to be active every day, thus maintaining the strength of the back muscles. Thus, this may explain the insignificant difference of the back muscle strength in the older males with and without LBP. Besides natural consequences of aging that could decrease muscle strength [38], another possible factor for women is menopause, which causes deficits in estrogen levels. The reduction in estrogen levels causes decreased bone mass density and muscle mass [39], and decreased muscle mass results in the loss of muscle strength [40]. In contrast, males have greater muscle mass than females due to testosterone, whose peak level occurs during puberty [41]. The author also stated that males had prolonged lifetime loss of muscle mass and strength. The combination of pain, muscle mass, and hormonal changes factors may hasten the reduction of muscle strength in females with LBP.

Hodges and Richardson [42] revealed that the contraction of TrA was delayed in subjects with LBP compared with those without LBP. O'Sullivan et al. [43] suggested the presence of a neuromuscular dysfunction in the abdominal muscle in patients with chronic LBP. This might explain how the muscle control of TrA was affected by the occurrence of LBP that could alter muscle control of TrA. On the other hand, the present study's findings demonstrated that muscle control of TrA and multifidus did not show significant differences between older persons with and without LBP. In fact, older persons with LBP had better muscle control of TrA and multifidus than older persons without LBP. This could be due to the testing positions used: supine and prone lying positions were implemented for the test, which were easy to perform, because the muscles were in a relaxed state. Even though the PBU test in supine position is commonly used and well established [44], tests in other positions, such as standing, are more challenging, because the muscles contract against gravity and might be less suitable and less stable for older persons. Therefore, if the test was conducted in a different position, the results might differ.

### 4.3. Correlation of Muscle Functions and Functional Performances

Another aim of this study is to determine the association between muscle functions and functional performances in older persons with LBP. This study discovered that muscle functions were associated with several functional performances among older persons with LBP.

According to another study, the impairment of back extensor strength leads to the motor and sensory deficit that affects balance performance [45]. Back extensor strength is an important contributor to walking endurance in obese older adults with LBP [46]. In addition, Suri et al. [47] found that trunk extension strength was associated with mobility and balance in older adults. Of note, back muscle strength might predict functional mobility during walking tasks. However, the present study revealed no correlation between back and abdominal muscle strength and balance, as well as mobility. This was probably due to adaptation among the subjects, as they walk around the residence to attend special occasions, religious ceremonies, and daily meals at the cafeteria which is far from their dorms.

We discovered a significant and moderate correlation between back muscle strength and lower limb function in the male group but not in the female group. It is interesting to note that the current finding is consistent with the study of Hicks et al. [48] that found that trunk muscle attenuation is associated with the chair stand performances among older persons. Muscle attenuation (lower fat infiltration) was also linked to reduced muscle strength. This might explain why trunk muscle strength was relatively associated with lower limb function. Back muscle strength also appeared to be important for lower limb function among older persons with LBP.

The present study also revealed that back and abdominal muscle strengths were associated with hand grip strength in the female group but not in the male group. Rantanen et al. [49] found that hand grip strength is associated with long-term mortality risk and can predict functional reserve that protects against mortality. More recently, Shahida et al. [50] stated that hand grip strength was a reliable factor reflecting the overall strength of older persons, as well as a good predictor of functional limitations and disability in older persons.

The TrA allows functional movements by stabilizing every segment of the lumbopelvic region when supporting the body's weight [14]. Stable and strong core muscles contribute to more efficient use of the upper and lower limbs and are important for the successful performance of day-to-day activities during old age [51]. The present study demonstrated that muscle control of the TrA was not correlated with all domains of functional performance in both male and female groups. During walking and chair rise tasks, core muscles are associated with and contribute to the execution of these tasks. However, in the study found muscle control of the TrA and multifidus was not associated with balance and mobility in both groups. The muscle control of TrA and multifidus was tested in prone and supine lying positions, respectively. Jung et al. [52] stated that the assessment of TrA using PBU in a standing position produced more significant increase in the activities of the muscles than supine position, because the muscles also overcame the gravity acting on them. The authors also highlighted the possibility that the contraction of TrA in a standing position is stronger than in a supine position in order to maintain body balance while standing, which likely helped in mobility during the TUG test. In future studies, we suggest the assessment of TrA and multifidus using PBU in a standing position to provide different results.

This study found that muscle control of the TrA and multifidus was not correlated with hand grip strength in both groups. TrA and multifidus are part of core muscles that initiate all limb movements, including hand movement. Miyake et al. [53] suggested that core stability exercise improved trunk stability, which might enhance shoulder and distal stability of the upper limb. The stability of both the shoulder and the distal parts of the upper limb improved the movement of the elbow and fingers. Thus, it may enhance the performance of the hand grip during execution of a task. However, the core stability exercise only improved neuromuscular facilitation but did not improve strength. This might reflect on the current findings that muscle control of the TrA and multifidus was not associated with hand grip strength, even though these muscles aid in hand grip function.

In the current study, back and abdominal strengths were associated with hand grip strength in females with LBP, and back strength and multifidus control were associated with lower limb function in males and females with LBP, respectively. Additionally, muscle strength of the back and abdomen predicted hand grip strength in older females with LBP. Subsequently, multifidus control predicted lower limb functions in older females with LBP. However, muscle functions did not predict functional performances in the male older persons with LBP. Therefore, it can be concluded that only parts of muscle functions predicted functional performances in older persons with LBP. The findings of the current study might be explained in this way. It is possible that functional performances among older persons with LBP predicted other variables. Ledoux et al. [54] revealed that functional capacity in older persons with LBP was dependent on physical activity and disability level. However, our study did not measure both outcomes objectively and thus those factors might be taken into account in order to predict functional performances in future study.

Furthermore, muscle functions and functional performances also are not significant predictors of pain intensity in older persons with LBP. Several factors have been recognized as risk factors of LBP including physiological factors, psychological factors, and social factors [3]. There were possibilities that the pain level in older persons with LBP was affected by psychological factors and social factors. In this study, we focused on evaluating physiological outcomes which include muscle functions and functional performances. In future, other factors need to be evaluated to determine the main predictors of pain intensity for older persons with LBP.

### 4.4. Study Limitations

This study had several limitations. First, we categorized the participants of this study into LBP and non-LBP groups; the LBP group was a mixed group of older persons with chronic and acute or subacute pain. The muscle function and functional performance may differ according to the duration of pain. The study also excluded many cases of outliers, whose scores were out of the score range of subjects. Moreover, for functional performance, only hand grip strength, TUG, and 30-Second Chair Rise test were measured. It is best if other outcome measures such as static and dynamic balance can be measured. Despite these limitations, to our knowledge, this paper presents the first study that evaluated the association between muscle functions and functional performances for older persons with LBP.

## 5. Conclusion

In conclusion, this study demonstrates that the functional performances in the older persons with LBP were not significantly different to older persons without LBP. For muscle functions, the older females with LBP had poorer abdominal and back muscle strength compared to older females without LBP, but not in the males. Muscle functions also were associated with functional performance in older persons with LBP. Additionally, muscle functions also predicted the functional performances in the older females with LBP. However, muscle functions did not predict functional performances in the older males with LBP. Hence, further studies should be conducted in the future, probably with different outcome measures, to validate the effects of muscle functions on functional performances in older persons with LBP.

## Figures and Tables

**Table 1:** Demographic characteristics, pain intensity, functional performances, and muscle functions of the subjects (*n* = 95).

Characteristics	LBP (*n* = 64)		NLBP (*n* = 31)		ANOVA		Post hoc	
	Male (A)	Female (B)	Male (C)	Female (D)	*p* value	Cohen's *d*	*p* value	Cohen's *d*
	(*n* = 34)	(*n* = 30)	(*N* = 18)	(*N* = 13)				
	Mean ± SD	Mean ± SD	Mean ± SD	Mean ± SD				
Age (years)	69.94 ± 8.38	71.87 ± 7.38	68.54 ± 4.22	70.27 ± 7.26	0.49	0.03	—	—
BMI (kg/m^2^)	23.04 ± 3.52	24.49 ± 4.71	22.05 ± 3.79	24.39 ± 4.02	0.17	0.05	—	—
Waist circumference (cm)	88.04 ± 10.84	87.73 ± 10.56	88.38 ± 12.49	92.77 ± 8.25	0.53	0.03	—	—
Body fat compositions (%)	29.69 ± 6.30	35.59 ± 6.17	30.30 ± 6.08	34.52 ± 5.39	0.00	0.17	AC = 1.000	0.098
							BD = 1.000	−0.18
Pain intensity	4.12 ± 1.59	4.13 ± 1.91	0 ± 0	0 ± 0	0.00	0.64	^**∗**^ **A** **C** = 0.00	−3.19
							^**∗**^ **B** **D** = 0.00	−2.571
Lower limb function (reps)	9.50 ± 3.56	9.07 ± 2.75	11.92 ± 3.79	9.76 ± 3.85	0.04	0.09	AC = 1.00	0.665
							BD = 0.718	0.222
TUG (s)	12.90 ± 4.98	14.12 ± 4.63	9.98 ± 2.15	11.96 ± 2.31	0.01	0.11	AC = 0.110	−0.689
							BD = 0.740	−0.528
Right hand grip strength (kg)	20.49 ± 7.22	13.92 ± 5.40	20.62 ± 6.39	16.33 ± 6.65	0.00	0.19	AC = 1.00	0.019
							BD = 1.00	0.416
Left hand grip strength (kg)	18.84 ± 7.68	13.20 ± 4.63	17.71 ± 6.86	15.57 ± 5.20	0.00	0.13	AC = 1.00	−0.152
							BD = 0.718	0.493
Abdominal strength (kg)	0.35 ± 0.05	0.33 ± 0.06	0.34 ± 0.06	0.39 ± 0.04	0.01	0.09	AC = 1.00	−0.187
							^**∗**^ **B** **D** = 0.006	1.093
Back muscle strength (kg)	0.35 ± 0.05	0.32 ± 0.04	0.36 ± 0.05	0.37 ± 0.04	0.00	0.15	AC = 1.00	0.02
							^**∗**^ **B** **D** = 0.007	1.25
TrA (mmHg)	69.02 ± 3.00	69.27 ± 2.92	69.44 ± 70.74	70.74 ± 2.18	0.30	0.04	—	—
Multifidus control (mmHg)	40.37 ± 2.12	40.37 ± 2.86	40.33 ± 1.65	42.18 ± 3.42	0.13	0.06	—	—

Comparisons were tested using one-way analysis of variance (ANOVA).

^*∗*^The mean difference is significant at the level of 0.05.

**Table 2:** Pearson's correlation coefficient of functional performances and muscle functions among genders in LBP group (*n* = 64).

Correlates	Male (*n* = 34)			Female (*n* = 30)		
	TUG	Lower limb function	Hand grip strength	TUG	Lower limb function	Hand grip strength
	*r*	*r*	*r*	*r*	*r*	*r*
	*p* value	*p* value	*p* value	*p* value	*p* value	*p* value
Abdominal strength	0.037	0.223	−0.037	0.073	0.100	^**∗**^0.377
	0.834	0.205	0.834	0.703	0.598	**0.040**
Back strength	−0.096	^**∗**^0.393	0.137	0.078	−0.006	^**∗**^0.396
	0.590	**0.022**	0.438	0.684	0.975	**0.030**
TrA control	0.009	−0.068	0.029	−0.046	0.104	0.044
	0.961	0.703	0.870	0.808	0.585	0.816
Multifidus control	0.009	−0.065	−0.100	0.077	^**∗**^0.363	0.002
	0.961	0.716	0.575	0.969	**0.049**	0.990

Comparisons were tested using Pearson's correlation coefficient analysis.

^*∗*^Correlation is significant at the level of 0.05 (1-tailed).

**Table 3:** Multiple linear regression of functional performances and muscle functions.

Dependent variables	Male					Female				
	Unstandardized		Standardized	*r* ^2^	*p* value	Unstandardized		Standardized	*r* ^2^	*p* value
	coefficients		coefficients			coefficients		coefficients		
	*B*	SE *B*	*β*			*B*	SE *B*	*β*		
Hand grip strength										
Constant	26.834	35.723		0.057		6.330	21.588		0.301	
Abdominal strength	−11.554	27.732	−0.082		0.680	35.972	16.702	0.410		^**∗**^0.041
Back strength	30.329	27.349	0.223		0.277	49.427	23.883	0.368		^**∗**^0.049
TrA control	0.216	0.500	0.090		0.669	0.054	0.387	0.029		0.890
Multifidus control	−0.691	0.718	−0.204		0.344	−0.584	0.428	−0.310		0.184
Lower limb function										
Constant	4.372	16.529		0.168		2.723	12.011		0.165	
Abdominal strength	6.314	12.831	0.091		0.626	−2.951	9.293	−0.066		0.753
Back strength	25.118	12.654	0.374		0.057	−6.807	13.288	−0.100		0.613
TrA control	−0.055	0.232	−0.046		0.814	−0.153	0.215	−0.163		0.483
Multifidus control	−0.052	0.332	−0.031		0.877	0.498	0.238	0.519		^**∗**^0.047
TUG										
Constant	4.160	24.902		0.039		18.813	21.978		0.015	
Abdominal strength	7.560	19.331	0.078		0.699	5.503	17.003	0.073		0.749
Back strength	−16.352	19.064	−0.174		0.398	8.989	24.314	0.078		0.715
TrA control	−0.073	0.349	−0.044		0.836	−0.138	0.394	−0.087		0.730
Multifidus control	0.419	0.501	0.179		0.410	0.005	0.435	0.003		0.992

Comparisons were tested using multiple linear regression.

^*∗*^The  *p* value is significant at the level of 0.05 (1-tailed).

**Table 4:** Multiple linear regression of pain, muscle functions, and functional performances.

Dependent variables	Male					Female					
	Unstandardized		Standardized	*r* ^2^	*p* value	Unstandardized		Standardized		*r* ^2^	*p* value
	coefficients		coefficients			coefficients		coefficients			
	*B*	SE *B*	*β*			*B*	*r* ^2^		*β*		
							*p*				
Pain											
Constant	16.065	7.662		0.224		0.601	8.705			0.284	
Abdominal strength	−10.949	6.135	−0.354		0.086	4.194	10.213	0.135			0.685
Back strength	−5.137	6.317	−0.171		0.423	4.105	8.335	0.171			0.627
TrA control	−0.053	0.107	−0.100		0.627	−0.056	0.155	−0.085			0.722
Multifidus control	−0.052	0.159	−0.069		0.747	0.066	0.199	0.098			0.745
Lower limb function	0.067	0.155	0.155		0.667	−0.030	0.215	−0.043			0.891
TUG	−0.031	0.098	0.098		0.757	0.047	0.118	0.115			0.692
Hand grip strength	−0.042	0.043	0.043		0.341	−0.030	0.215	0.324			149

Comparisons were tested using multiple linear regression.

^*∗*^The  *p* value is significant at the level of 0.05 (1-tailed).
